# Do No Harm: Considerations for Pediatric Orthopaedic Practice During the Coronavirus Pandemic

**DOI:** 10.5435/JAAOSGlobal-D-20-00055

**Published:** 2020-05-14

**Authors:** Emily K. Schaeffer, Jeffrey N. Bone, Kishore Mulpuri

**Affiliations:** From the Department of Orthopaedics, University of British Columbia, Vancouver, British Columbia, Canada (Dr. Schaeffer and Dr. Mulpuri); the Department of Orthopaedic Surgery, BC Children's Hospital, Vancouver, British Columbia, Canada (Dr. Schaeffer and Dr. Mulpuri); and the Department of Obstetrics and Gynaecology, University of British Columbia, Vancouver, British Columbia, Canada (Mr. Bone).

On March 11, 2020, the World Health Organization (WHO) officially declared the coronavirus (COVID19 or SARS-CoV-2) epidemic as a pandemic, after previously declaring it a Public Health Emergency of International Concern on January 30, 2020.^1^ First reported as a cluster outbreak in Wuhan, China, in December 2019, it has now spread through 205 countries at the time of publication.^1^ The number of global cases and deaths due to SARS-CoV-2 are escalating with alarming speed, as countries worldwide take unprecedented measures in attempts to control the spread.

Our knowledge of viral transmission, disease course, characteristic symptoms, and potential treatments for those severely impacted is rapidly evolving as the pandemic continues to progress.

British Columbia currently has the second most SARS-CoV-2 cases in provinces and territories across Canada. As of April 3, 2020, there are a total of 1,174 confirmed cases, with 35 deaths and 146 currently in hospital, 64 of whom are currently in intensive care. Public health officials and healthcare workers have mobilized attempts province wide to limit the spread of SARS-CoV-2, issuing stay-at-home recommendations for all nonessential workers, closure of nonessential businesses, and levying fines for failure to practice social distancing.^2^

SARS-CoV-2 is primarily transmitted from person to person through respiratory droplets generated when an infected person sneezes or coughs. Early estimates from Chinese data suggest that an average of 3.02 (95% CI, 2.65–3.39) people can become infected via transmission from a viral carrier.^3^ Consequently, social distancing is one of the most critical and effective practices to limit the spread of the virus. Limiting potential spread is of crucial importance in the healthcare setting, where healthcare providers can come in contact, conservatively, with hundreds of patients, family members, and other healthcare providers each day. This is especially true in an extremely busy service such as pediatric orthopaedics. At BC Children's Hospital (BCCH), orthopaedic surgery is the busiest department in the hospital outside of the emergency department, seeing an estimated 14,000 patients each year.

Although the field of orthopaedic surgery does not immediately come to mind when considering frontline responders in the wake of the crisis, orthopaedic surgery and other specialties must play a critical role in reducing the spread of SARS-CoV-2 while balancing the needs of their patients. Of particular concern is asymptomatic healthcare workers unknowingly driving viral transmission to patients, families, and other healthcare workers in these high-contact environments. A report of Singapore comments on the early experiences of orthopaedic surgical management during the outbreak.^4^ As one of the first countries to experience the epidemic, this report can provide valuable insight to other orthopaedic units around the world still bracing treatment for the peak of the outbreak. The guiding principles for the Singaporean orthopaedic service were clinical urgency, patient and healthcare worker protection, and conservation of healthcare resources. Although musculoskeletal trauma and tumor surgeries continued as much to schedule as possible, most elective cases were indefinitely postponed, particularly those cases that would typically require overnight or longer stays in hospital postsurgery. Similarly, the British Orthopaedic Association recently released guidelines emphasizing the management of patients with nonoperative strategies and minimizing outpatient clinic visits.^5^ Recommendations for the management of pediatric fractures have also echoed these precautions.^6^

Taking these early experiences from Singapore, the United Kingdom and other countries into consideration, many orthopaedic units in hospitals across the globe have begun to take preventive measures by reducing or closing clinics to most outpatients. The BCCH Orthopaedic Surgery department has scaled back clinics as much as possible and has made key decisions to limit or postpone treating children with orthopaedic conditions such as developmental dysplasia of the hip (DDH) for potentially the next 3 months. Balancing the effort to limit SARS-CoV-2 transmission, the need to keep patients and healthcare workers safe, and the long-term impact on patient quality of life with delayed orthopaedic treatment is a challenging task and highlights the importance of assessing the risks and benefits to all involved.

When considering pediatric patients with congenital, developmental, or other chronic orthopaedic issues, the decision to defer clinic visits for up to 3 months may be fraught with concern over the long-term impact of delayed treatment. This is particularly true in conditions such as DDH, where early detection and treatment are widely regarded to optimize outcomes. Specifically, a 2016 study suggested that the initiation of treatment after 7 weeks was associated with an increased risk of failure of conservative approaches.^7^ Patients with DDH account for a large volume of clinic visits and require contact with a number of different care providers. Below, we use DDH as an example to demonstrate the potential number of contacts between patients/families and care providers that can be avoided with clinic closure, and why long-term impact of delayed treatment may not be of substantial concern.

The BCCH Orthopaedic Clinic typically will see around 25 infants per week referred for possible or suspected DDH across all staff surgeons. Although most of these infants will not require further treatment, approximately 5% of these infants will be diagnosed with hip dislocations or some degree of dysplasia. These children will then also require regular follow-up, whether weekly, biweekly, or at minimum a 6-week check-up. An additional 5% of these infants, who may not require immediate treatment, may qualify for a “watch and wait” approach with mild dysplasia and/or slight hip instability. These infants would thus typically return to clinic for a 6-week follow-up visit. It is not uncommon for at least 2 to 3 family members to accompany the infant to the hospital per visit. During an initial DDH assessment, patients and families are likely to come into contact with a minimum of four care providers in different clinical areas of the hospital: a registration clerk, an ultrasonography technician, a clinical staff member or nurse, and the orthopaedic surgeon or clinical fellow. Under normal high capacity clinic conditions, a minimum of five care providers are involved, as a resident/fellow/medical student will typically examine the patient along with the orthopaedic surgeon.

Table 1 provides projections for estimated contacts between care providers and patients and families for low-, moderate-, and high-case scenarios. The low-case scenario assumes that while the orthopaedic clinic continues to run, accompanying individuals have been limited to one parent/guardian per patient, contact with care providers per visit has been limited to two and follow-up visits within the 2-month period are limited to two. The moderate-case scenario represents a typical clinic with slight limitations on accompanying family members. In both low- and moderate-case scenarios, 6-week follow-up visits for “watch and wait” patients have been eliminated. The high-case scenario represents continuing to run as normal with no restrictions.

**Table 1:** Conservative, Moderate, and Regular Contact Scenarios in the Orthopaedic Clinic

	Conservative	Moderate	Regular Clinic
No. of children per week	25	25	25
Average number adults accompanying/child	1	2	2.5
Rate needing full follow-up	5%	5%	5%
Average number of follow-up for above	2	4	4
Rate needing 6-wk follow-up	5%	5%	10%
No. of clinic staff interacted with	2	4	5
Projected number of total interactions per week (for all staff)^a^	115	375	569
Projected number of total interactions in the 12-wk period	1380	4500	6828

a Does not account for any staff-to-staff interaction.

It is evident that a substantial number of interactions between patients with DDH, families, and care providers would occur over a 12-week period even if the orthopaedic clinics were to operate with significant restrictions in place (1,380). If no restrictions were implemented, an estimated 6828 interactions would occur, without accounting for any staff-to-staff interactions. Although initial impressions of SARS-CoV-2 have been that children are not as susceptible to infection, a recent retrospective review of the epidemiology of 2143 pediatric cases in China suggested that infants were more susceptible to the severe form than older children.^8^ Specifically, 10.6% of cases were severe or critical in infants younger than one year of age, compared with 3.0% of cases in adolescents between 16 and 18 years.^8^ Given that most children coming to the orthopaedic clinic for DDH are younger than one year of age, these patients represent a potentially vulnerable population to SARS-CoV-2 infection.

Delaying clinics for the diagnosis and treatment of children with DDH has the potential to greatly reduce the number of potential SARS-CoV-2 transmissions between patients/families and care providers. Considering the cross-disciplinary nature of care providers in DDH (orthopaedics, radiology), delay of these clinics could also aid in preventing the spread of SARS-CoV-2 across different healthcare units within the hospital. Substantial consideration, however, must be given to balancing appropriate and timely clinical care for children with DDH. When caught early, conservative treatment with a harness or brace is often successful, even in the cases of complete dislocation. In the cases of late presentation or late detection, surgical treatments such as closed or open reduction may be necessary. Although a decision to defer these patients for 3 months does introduce concerns about late detection, data from the International Hip Dysplasia Registry (IHDR) suggest that harness or brace treatment can still be effective in older infants and that if necessary, closed reduction is successful in 91% of cases in infants up to one year of age.^7,9^ Although an untreated ortolani-positive hip may eventually require open reduction, the success rates of both closed and open reduction even in older infants seen within International Hip Dysplasia Registry can allay some of these concerns in the current climate. Consequently, the closure of the orthopaedic clinic to these patients for a 3-month period at the peak of the pandemic should likely have minimal impact on the long-term orthopaedic outcomes for the patient.

We would recommend other pediatric orthopaedic units in SARS-CoV-2 affected areas to consider temporary clinic closures, particularly in hospitals where personal protective equipment (PPE) is in limited supply. A survey of orthopaedic surgeons in Wuhan, China, working during the initial outbreak found that 26 surgeons in eight hospitals across Wuhan tested positive for SARS-CoV-2, emphasizing the need for adequate PPE to protect care providers, patients, and families.^10^ Should proper supply of PPE become available, clinics may consider reopening for less urgent but time-sensitive orthopaedic management.

Balancing health and safety of patients, families, and care providers with appropriate timely orthopaedic care during the SARS-CoV-2 crisis is of utmost importance. Pediatric orthopaedic clinics can substantially contribute to the efforts to slow the spread of infection by taking early preventive action with clinic closures to nonurgent patients without compromising patient care. These actions could also likely be applied in other specialties, particularly those with high-risk patients, aiding to reduce unnecessary illness or death from SARS-CoV-2.
